# Assessment of greenness, blueness, and whiteness profiles of a validated HPLC-DAD method for quantitation of Donepezil HCl and Curcumin in their laboratory prepared co-formulated nanoliposomes

**DOI:** 10.1186/s13065-024-01377-y

**Published:** 2025-01-18

**Authors:** Mennah M. Abd Elwahab, Samar Saad, Zeinab A. Sheribah, Nahed El-Enany

**Affiliations:** 1 Department of Pharmaceutical Analytical Chemistry, Faculty of Pharmacy, Sinai University - Arish branch, Arish, 45511 Egypt; 2 Department of Pharmaceutical Analytical Chemistry, Faculty of Pharmacy, Mansoura University, Mansoura, 35516 Egypt; 3 Department of Pharmaceutical Analytical Chemistry, Faculty of Pharmacy, New Mansoura University, New Mansoura, 7723730 Egypt

**Keywords:** Donepezil HCl, Curcumin, HPLC-DAD, Nanoliposomes, Greenness, Whiteness

## Abstract

White and Green Analytical Chemistry are innovative approaches in analytical chemistry that prioritize both sustainability and efficiency. Together, these approaches aim to advance scientific research while minimizing environmental impact and enhancing safety. This integration of environmental consciousness into analytical practices represents a significant step forward in achieving sustainable scientific progress. In the present study, a sensitive eco-friendly HPLC-DAD method was carried out and validated to allow concurrent determination of Donepezil HCl (DPZ) and Curcumin (CUR) in their pure form and laboratory made nano-liposome formulation. Optimum seperation was accomplished by utilising Zobrax Eclipse Plus C18 column (4.6*100 mm,5 μm) with gradient elution of the mobile phase composed of 0.02 M phosphate buffer at pH 3.2 and ethanol at flow rate of 1.5 ml/min. A diode array detector (DAD) was implemented for detection at 273 nm and 435 nm for DPZ and CUR, respectively, with the column oven set at 40 °C. The method was validated according to ICH specifications in terms of accuracy, precision, linearity range, detection and quantification limit. The calibration plots were linear with correlation coefficients (r2) = 0.999 over the range (0.1–100 µg/ml) and (0.1–100 µg/ml) for DPZ and CUR, successively. The validated HPLC-DAD approach was adopted to analyse both medications in laboratory prepared nano-liposomal formulation in which the analytes were successfully quantified with good recovery values and no disrubtion from the added excipients. The investigation of whiteness, blueness, and greenness metrics revealed a major benefit of the suggested approach over previous reported ones.

**Supplementary Information:**

The online version contains supplementary material available at 10.1186/s13065-024-01377-y.

## Introduction

Alzheimer’s disease [AD] is a chronic progressive neurodegenerative disorder characterized by intellectual disability, amnesia, behavioural and personality changes. Pathologically, AD is distinguished by Prescence of β-amyloid plaques extracellular and neurofibrillary tangles [NFTs] intracellular in the brain [1]. The typically recommended drugs for AD are only symptomatic treatment, they do not halt the disease’s degenerative pathology. Among the treatment strategies used for symptomatic treatment of AD is to enhance the cholinergic activity in the brain by blocking the enzyme which is responsible for breaking down Acetylcholine neurotransmitter [2].

**Fig. 1:**
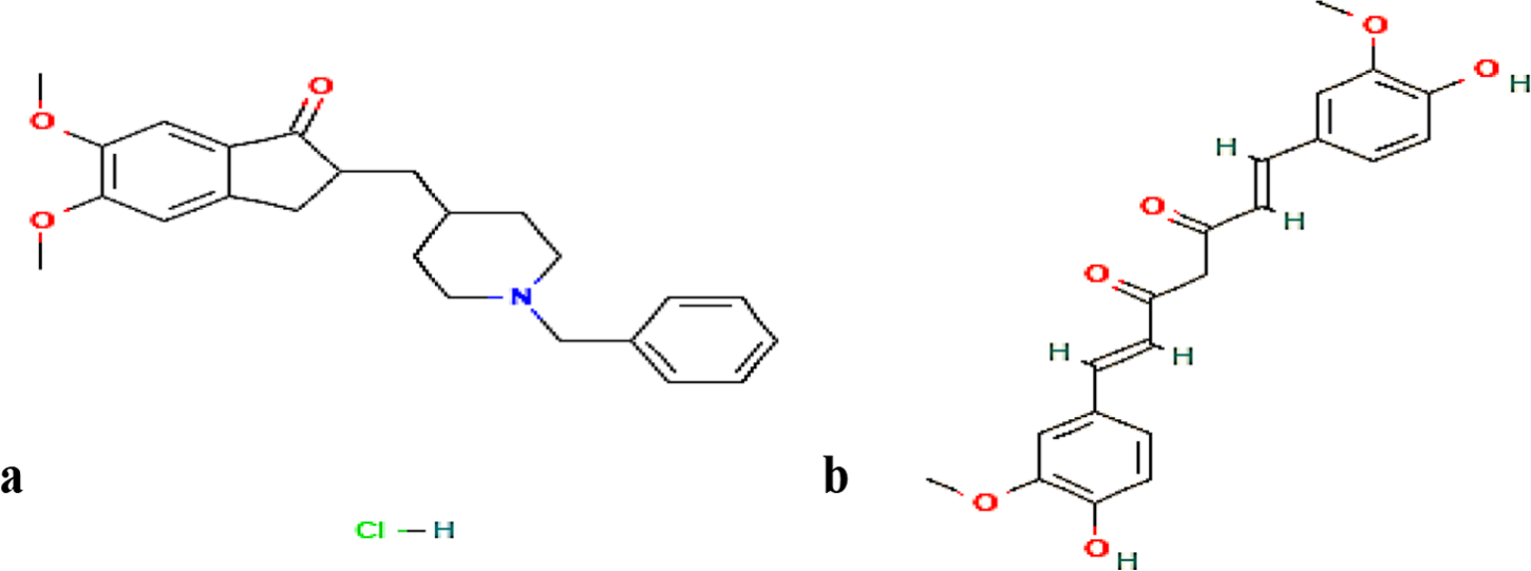
Chemical structures of (**a**) DPZ and (**b**) CUR

Donepezil hydrochloride (DPZ) ;2-((1-Benzylpiperidin-4-yl) methyl)-5,6-dimethoxy-2,3-dihydro-1 H-inden-1-one [3]; Fig. 1a, is a white powder that is insoluble in n-hexane, very hardly soluble in ethyl acetate, barely soluble in acetonitrile, sparingly soluble in ethanol and glacial acetic acid and readily soluble in water and chloroform [3]. It is the second drug given approval by the FDA in 1996 for mild to moderate AD treatment, which was subsequently authorized in 2010 for moderate to severe AD at a dose of 23 mg/day [2]. It is a widely used member among the cholinesterase inhibitors group which can’t cure AD nor stop the disease progression but it’s a symptomatic treatment which improves the cognitive ability and thinking in patients [4]. HPLC-UV assay was employed in the official USP 38 monograph to determine DPZ in sample and standard solutions at 271 nm using a mobile phase composed of acetonitrile and buffer adjusted at pH 1.8 using perchloric acid in the ratio (35:65) [5]. Different spectroscopic methods were published for DPZ estimation suffering from low sensitivity [6, 7]. Different HPLC methods either with UV or fluorescence detection have been published for determination of DPZ [8–11]. Additionally, HPLC methods hyphenated with tandem mass spectrometry were published for determination of DPZ which have suffered from difficult extraction methods for the drug from biological fluids. Also, HPLC-MS/MS is an expensive technique that is not available at all quality control laboratories [12].

Curcumin (CUR); (1E,6E)-1,7-bis(4-hydroxy-3-methoxyphenyl)hepta-1,6-diene-3,5-dione [13]; Fig. 1b, has been found to be effective in prevention, diagnosis and treatment of AD. Also, its native fluorescence and high binding affinity to β-amyloids make it a useful diagnostic tool for AD. It’s a co-administered antioxidant and anti-inflammatory given with DPZ for protection of the cerebral vessels, prevention and treatment of AD [14]. According to a literature review, it was found that many UV-spectrophotometric methods have been published for determination of CUR [15–17]. Also, many HPLC methods have been published for estimation of CUR in biological fluids and different dosage forms either individually or in combination with other drugs [18–22].

It was found that the pharmaceutical combination of DPZ and CUR enhances memory and cognitive ability and has a notable Acetylcholinesterase (AchE) and Butyrylcholinesterase (BchE) inhibitory effect [23, 24]. CUR has poor bioavailability when administered orally due to its rapid metabolism in the liver and intestine. Nanoliposomes are spherical bilayer vesicles with aqueous core encapsulating the hydrophilic drug and phospholipid bilayer loaded with the hydrophobic drug. CUR loaded nanoliposomes protect it from early degradation in the gastrointestinal tract which improves its absorption and bioavailability. Additionally, this allows for the sustained and controlled release of CUR over time [25]. Also, CUR is prone to degradation in physiological conditions, particularly when exposed to light, heat, or oxidation, encapsulating it in nanoliposomes offers protection from environmental factors, thereby increasing its stability and shelf life [26]. Moreover, it is poorly soluble in water, which limits its use in aqueous environments. Nanoliposomes provide a hydrophobic bilayer that accommodates lipophilic compounds like CUR, allowing it to be solubilized in biological fluids [27]. Therefore, formulating CUR as nanoliposomes overcomes several of the limitations associated with it’s natural form, such as poor bioavailability, instability, and low water solubility. The nanoliposomal formulation enhances it’s therapeutic potential by improving bioavailability, targeting specific tissues, allowing sustained release, and protecting it from degradation. This makes nanoliposomal CUR a promising candidate for a wide range of therapeutic applications, particularly in cancer, inflammation, and neurodegenerative diseases. On the other hand, DPZ undergoes non-targeted distribution when administred orally, which can lead to gastrointestinal adverse reactions like nausea, vomiting, diarrhoea, bleeding in the stomach, convulsions in the muscles, and insomnia. Because of its hydrophilicity, DPZ is poorly capable to cross the blood brain barrier (BBB) following oral administration. It also experiences hepatic first-pass metabolism [28]. By encapsulating DPZ in nanoliposomes, it is possible to deliver the drug more selectively to the brain while reducing its distribution to other parts of the body. This targeted delivery minimizes off-target effects and reduces the severity of systemic side effects [29]. All of these factors encouraged us to co-formulate CUR and DPZ in a laboratory prepared nanoliposome formulation which was selectively and precisily analysed by the proposed HPLC-DAD methodology.

The history of green chemistry and green analytical chemistry (GAC) reflects a growing awareness of the need for eco-friendly practices in the chemical sciences. Green Chemistry emerged in the late 20th century as a response to the environmental and health impacts of traditional chemical processes, driven by the recognition that chemistry could be both innovative and environmentally responsible [30, 31]. GAC evolved from green chemistry to focus specifically on developing analytical techniques that minimize environmental and health risks, such as reducing solvent use and waste. White analytical chemistry (WAC) is a recently established approach for assessing the efficiency of analytical procedures in terms of sample analysis, cost-effectiveness, and greenness strength. WAC is assessed using the red, green and blue colors which combine together forming the white color (RGB model) [32]. In keeping with sustainable environmental standards, the latest conceptions of blueness were implemented as well. A novel measurement tool called the blue applicability grade index (BAGI) has been put forth to assess an analytical method’s practicality. BAGI is primarily emphasising on the practical features of WAC and it may be viewed as a complement to the well-established green metrics [33]. Together, these fields represent a transformative shift in how chemistry is practiced, promoting sustainability and environmental stewardship throughout the chemical lifecycle.

To the best of our knowledge, only one method was published for simultaneous determination of both drugs in insitu nasal gel [34]. It is a stability indicating HPTLC method which was performed on pre-coated silica gel HPTLC plates and a mobile phase composed of toluene, methanol and glacial acetic acid in the concentration range (300–1800) and (20–720) ng/band for DPZ and CUR, respectively. HPTLC is a more sophisticated and automated version of TLC with improved separation, efficiency and detection limitations. On the other hand, HPTLC technique suffers from using bulky instrumentation, high costs, highly controlled operating circumstances (dust-free, temperature-controlled environments), and technically competent personnel with the experience to operate the system [35]. Although, HPLC is a costly process that requires several expensive organics and ongoing maintenance but it is an extremely precise technique which allows the use of gradient elution system during analysis. Because of its feasibility, broad availability, and reliability, HPLC is frequently utilized in conventional quality control laboratories [36]. The previously published method [34] suffered from utilizing organic solvents, which have a significant negative ecological consequences. This inspired us to establish and validate a white environmentally benign, cost-effective, time saving and reliable method for estimation of the co-administered mixture of DPZ and CUR in their pure form and in their laboratory prepared co-formulated nanoliposome formulation using HPLC-DAD technique with gradient elution of phosphate buffer (pH = 3.2) and ethanol as a mobile phase. The claimed greenness of the established method was approved using two greenness evaluation tools, Analytical GREEnness metric (AGREE) [37] and Modified Green Analytical Procedure Index (MoGAPI) [38]. In addition to being environmentally friendly, it is crucial for QC laboratories to employ procedures that fulfil the criteria required for efficiency and practicality. Consequently, the RGB algorithm was utilized for assessing analytical method whiteness [32]. Furthermore, contemporary conceptions of blueness were applied, aligning with the principles of sustainable environmental practices [33].

## Experimental

### Instrumentation and software

The liquid chromatography system was Hitachi LaChrom^®^ Elite (HITACHI High-Technologies Corporation – China) composed of a L-2455 photodiode array detector and L-2130 quaternary pump with a built-in degasser. The system was controlled, and peak areas were integrated automatically by computer using EZ LaChrome Elite software program. A 7725i Rheodyne injector system with a 20 µl sample loop was used for manual injection of samples. The separation was performed using Intersil^Ⓡ^ (Tokyo, Japan) ODS-3 column (5 μm, 250 × 4.6 mm; GL Sciences Inc.). pH adjustment was employed using Consort pH-meter Model P‐901 (Turnhout, Belgium).

### Reagents and materials

DPZ (99.7%) pure standard was kindly supplied by HIKMA PHARMA, Cairo, Egypt. CUR (99.0%) pure standard was provided by Sigma-Aldrich, Germany. Ortho phosphoric acid 85%, HPLC grade solvents ethanol and methanol were purchased from Sigma-Aldrich Co., (Steinheim am Albuch, Germany). Potassium dihydrogen orthophosphate was purchased from El Nasr pharmaceutical Co., Cairo, Egypt.

### Preparation of the nanoliposomal co-formulation

**Fig. 2:**
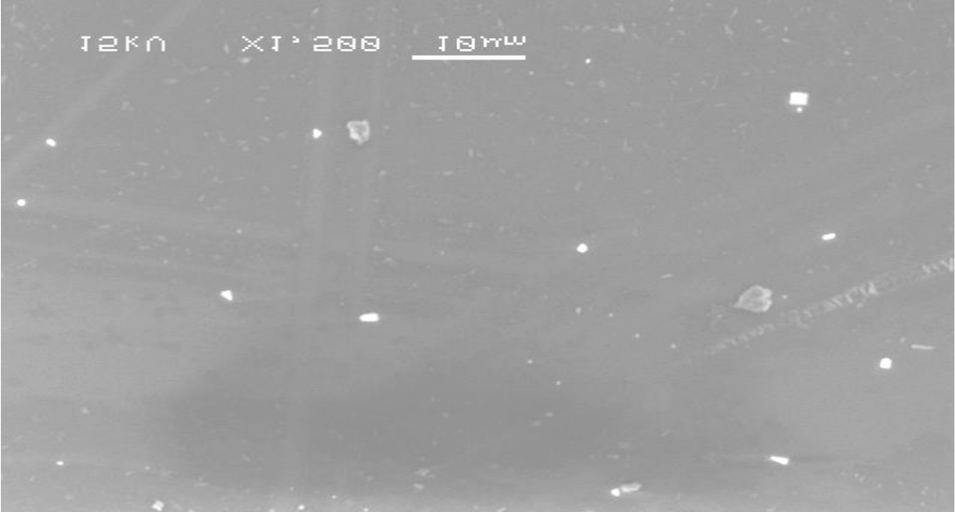
Microscopical examination of the laboratory prepared nanoliposomes

DPZ and CUR co-formulated nanoliposomes were prepared by thin film hydration method [39, 40]. Briefly In a round bottom flask, 10 mg of CUR (lipophilic drug), phospholipid ( L-alpha-phosphatidyl choline) and cholesterol (5-cholesten-3beta-ol) were added in equal ratio 1:1. The lipidic portion was dissolved in a mixture of chloroform and ethanol, then subjected to vacuum rotary evaporator. Solvents were evaporated leaving a thin film. The film was hydrated with distilled water containing 10 mg of DPZ under continuous rotation at 40 ⁰c. After the dispersion was obtained, it further subjected to water bath sonication for 30 min. Microscopical examination was done to confirm vesicles formation, as shown in Fig. 2.

### Preparation of buffer component of Mobile Phase

2.72 grammes of potassium dihydrogen phosphate were dissolved in 1 L of double distilled water. The pH was adjusted at 3.2 (± 0.05) with 85% orthophosphoric acid. The mobile phase buffer was filtered through a 0.45 𝜇m nylon membrane filter using a Millipore glass filter holder. The mobile phase buffer was used immediately after preparation or refrigerated in closed glass containers for up to 24 h.

### Standard stock solutions

Stock solution of 100 µg/ml for each DPZ and CUR was prepared in methanol. Serial dilutions using methanol were prepared from the standard stock solutions of each drug to attain working solutions in the range (0.1–100 µg/ml) and (0.1–100 µg/ml) for DPZ and CUR, respectively. When refrigerated at 4 °C, the prepared solutions retained stable for at least 10 days.

### Chromatographic conditions

To separate the binary mixture, a reversed phase C18 column (5 μm, 250 × 4.6 mm) and a mobile phase comprised of 0.02 M phosphate buffer adjusted at pH 3.2 and ethanol running at 1.5 ml/min were utilised. As illustrated in Table 1, gradient elution was accomplished at 1.5 ml/min flow rate and 20 µL injection volume at 40 °C. The analysis was conducted after a 15-minute column conditioning duration. For DPZ and CUR, the diode array detector’s λmax values have been adjusted at 273 nm and 435 nm, respectively.

**Table 1:** HPLC-DAD gradient elution program for analysis of DPZ and CUR

Time (min.)	Solvent A% (Buffer)	Solvent B% (Ethanol)	Flow rate (ml/ min.)
0	20	80	1.5
1	30	70	1.5
2	40	60	1.5
3	50	50	1.5
4	60	40	1.5
5	70	30	1.5
6	80	20	1.5
8	80	20	1.5

## Procedures

### Construction of calibration curve

Serial concentrations of the cited drugs were prepared by transferring different volumes from 100 µg/ml stock solutions into a set of 10 ml volumetric flasks and then completed to the mark with methanol to attain dilutions ranging from 0.1 to 100 µg/ml for DPZ and 0.1–100 µg/ml for CUR. Triplicate injections of each concentration of the tested drugs’ prepared solutions were conducted under optimal chromatographic conditions. The calibration curves and regression equations were derived by plotting each drug concentration in µg/ml versus its corresponding peak area.

### Assay of DPZ and CUR in synthetic mixtures

Aliquots were taken from the standard stock solution of each drug in different ratios and transferred to 10 ml volumetric flasks and completed to the mark with methanol, then analysis was performed following the proposed method as mentioned under “construction of calibration curve”.

### Assay of laboratory prepared nanoliposome formulation

Methanol was used as a solvent for extraction of the cited drugs from the prepared nanoliposomes. Each ml of the nanoliposomal formulation contains 1 mg of both DPZ and CUR, 100 µg/ml solution was prepared by transferring 1 ml of the preparation to 10 ml volumetric flask and diluted with methanol. The solution was vortexed for 10 min and sonicated for 30 min. Further dilution with methanol was carried out giving 10 µg/ml extract solution. The diluted solution was filtered through a 0.45 μm syringe filter before HPLC injection, and then analyzed as mentioned under the same chromatographic conditions. The results obtained as % recovery were compared to the amount of each drug used in nanoformulation preparation.

## Results and discussion

Achieving adequate resolution and acceptable peak symmetry in a suitable short analysis time while maintaining sustainability are the main targets of developing our HPLC-DAD approach. A reliable and precise HPLC method with gradient elution was developed and utilized successfully for separation of the co-administered anti-Alzheimer combination of DPZ and CUR.

### Method development and optimization

The optimal sensitivity of the medications under investigation has been established by adjusting the DAD at wavelengths of 273 nm for DPZ and 435 nm for CUR after recording the absorption spectra.

**Fig. 3:**
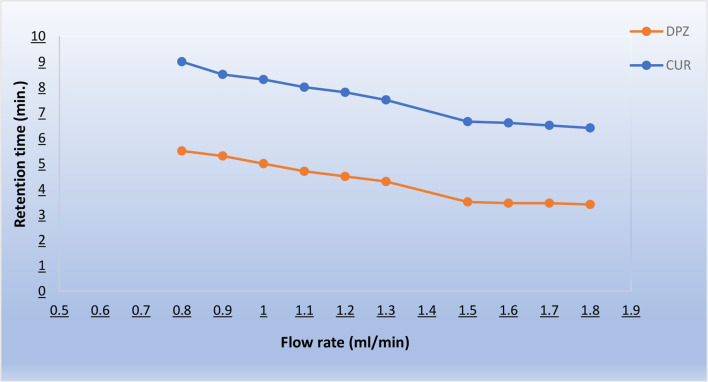
The effect of flow rate (ml/min) on the retention time of both separated drugs DPZ and CUR

The mobile phase composition plays a critical role in achieving a good chromatographic behavior. However, using large amounts of organic solvents and producing large quantities of waste are among the drawbacks of HPLC affecting the analyst safety and causing negative environmental impact [41]. Therefore, utilizing green solvents with benign ecological impact and waste minimization were our targets in this study. Varying proportions of aqueous phases and organic ones have been investigated to evaluate numerous mobile phase combinations. Several mixes of green solvents such as water, ethanol, methanol and buffers were tried in various ratios in an isocratic mode. The best resolution of the two drugs yielding sharp peaks in a suitable run time was achieved by the gradient elution of a mobile phase composed of ethanol as the organic modifier with potassium dihydrogen phosphate buffer adjusted at pH 3.2 as the aqueous component. At 37 °C, DPZ exhibits considerable solubility in the pH range of 1.2–6.8 [42], and it has a pKa value of 8.9 [43]. CUR has three distinct pKa values, the first pKa ranging from (7.7–8.5) and the second pKa from (8.5–10.4), these values result from the dissociation of the two phenolic protons. The third pKa value (9.5–10.7) comes from the dissociation of enolic proton under basic conditions [44]. CUR exists in a neutral keto-enol form under acidic pH. Also, CUR shows higher stability and less degradation as kept under acidic conditions at pH < 7 [44]. Therefore, acidic conditions were adopted using various ratios from potassium dihydrogen phosphate buffer adjusted at pH range 2.5–6.5 using diluted orthophosphoric acid in combination with ethanol. The best resolution was achieved using 0.02 M potassium dihydrogen phosphate buffer at pH 3.2 in combination with ethanol in a gradient elution manner starting from 20% ethanol ramped up linearly to 80% throughout the run in 6 min then kept at this percentage thereafter as shown in Table 1. Different flow rates ranging from (0.8–1.8 ml/min) were tried and their effect on the resolution of separation was studied. The best separation was achieved at suitable retention time with acceptable efficient sharp peaks at a flow rate of 1.5 ml/mim, as shown in Fig. 3.

**Fig. 4:**
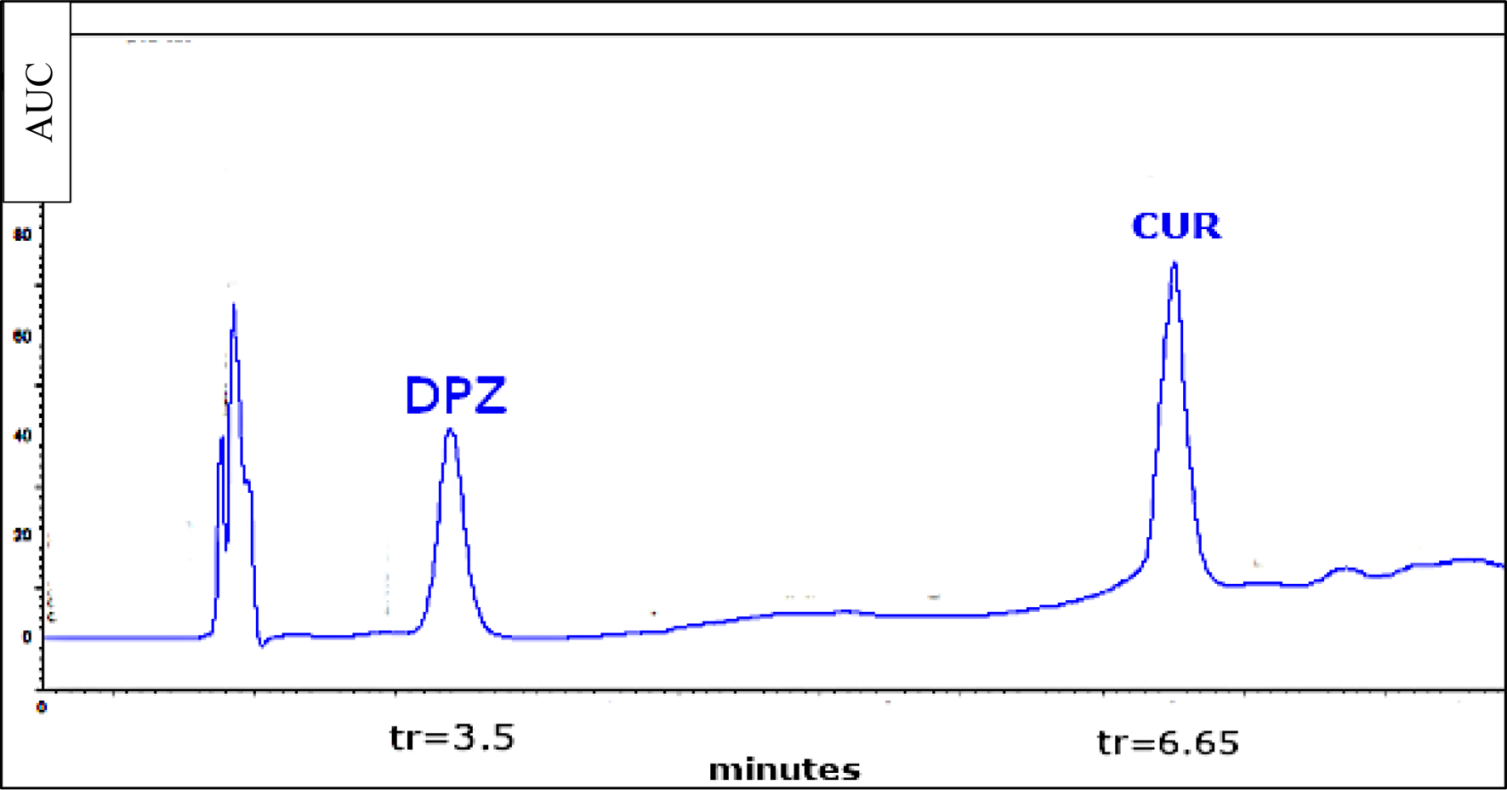
Representative HPLC-DAD chromatogram for separation of a standard mixture containing 5 µg/ml of both DPZ and CUR at 273 nm

Using the previously mentioned mobile phase and flow rate, some attainable columns have been examined in order to optimise the stationary phase, such as HyperClone™ MOS (C8) column (150 × 4.6 mm, 5 μm)and Intersil ODS-3 column (250 × 4.6 mm, 5 μm). The Intersil ODS-3 column exhibited the best resolution between the two drug peaks in a brief period of time, thereby it was recommended for this combination.These were found to be the most optimum conditions for the mixture separation with sufficient resolution in short run time. DPZ is ionized at acidic pH and was eluted first while the neutral CUR was retained for longer time, as illustrated in Fig. 4. The retention times of DPZ and CUR were observed at 3.6 ± 0.2 and 6.6 ± 0.2 min, respectively. The DAD gives the advantage of measuring each analyte at its maximum wavelength giving higher sensitivity.

### Method validation

The suggested method’s linearity, accuracy, precision, and robustness have been verified using the International Council for Harmonization’s ICH Q2 (R2) guidelines [45].

### Linearity and concentration ranges

**Fig. 5:**
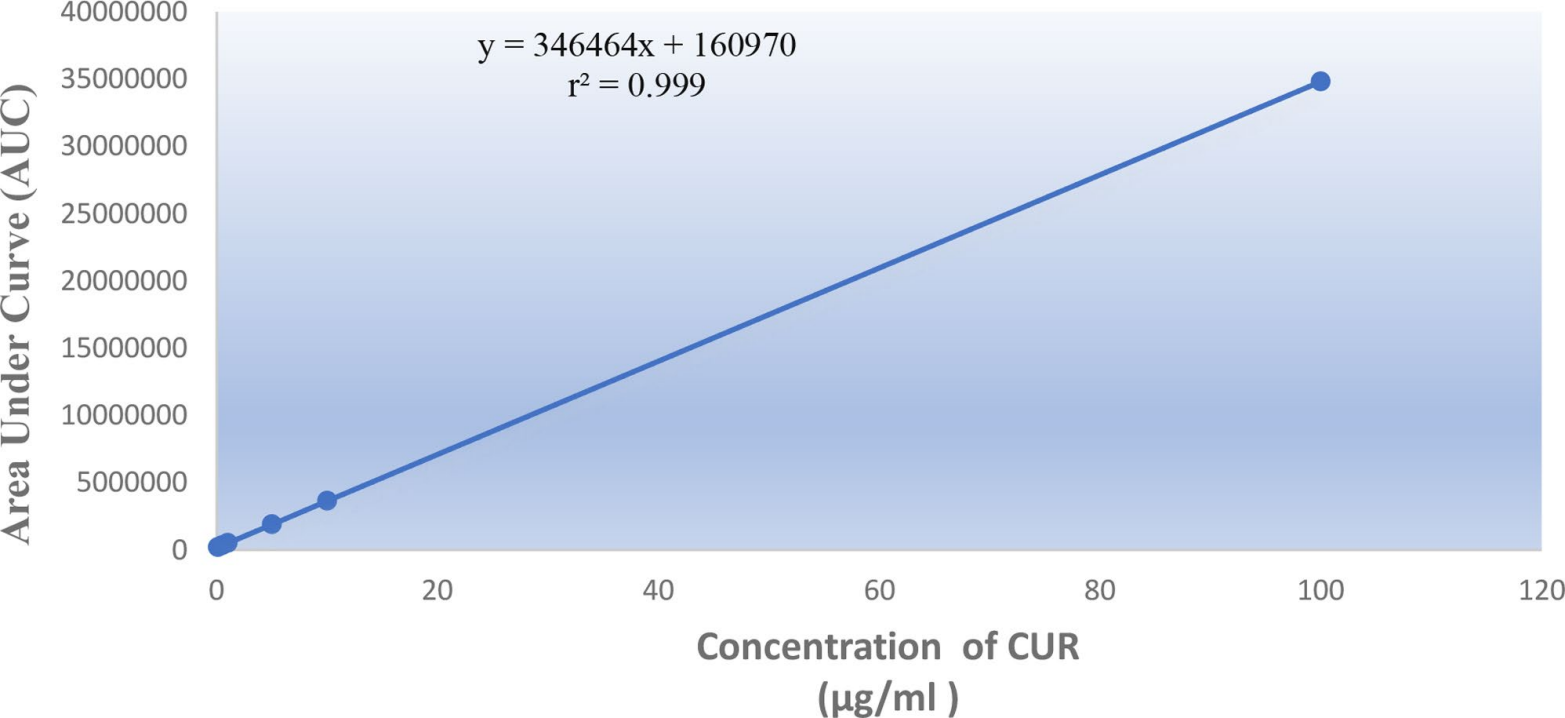
Standard calibration curve of (0.1–100 µg/ml) CUR by the proposed HPLC-DAD method

**Fig. 6:**
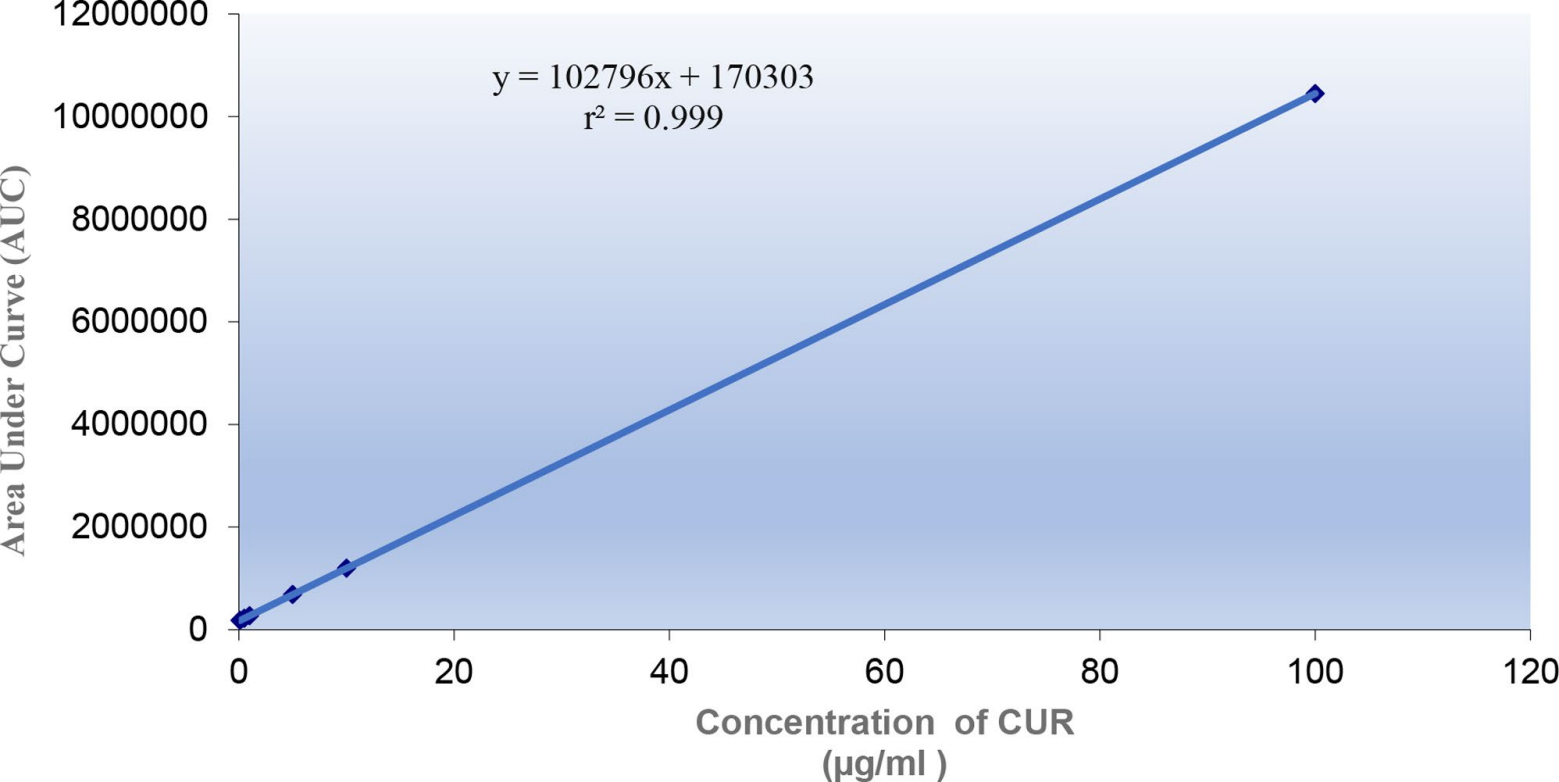
Standard calibration curve of (0.1–100 µg/ml) DPZ by the proposed HPLC-DAD method

To assess the linearity of the suggested HPLC-DAD methodology, a series of various concentrations have been investigated within predetermined ranges for each compound. Using the optimum chromatographic conditions, both CUR and DPZ were accurately determined in the range from 0.1 to 100 µg/ml, as presented in Figs. 5 and 6, respectively. It was found that there were linear relationships between the average peak areas and corresponding concentrations as demonstrated by the exceptional correlation coefficients values (r), as shown in Table 2.

**Table 2:** Analytical Performance Data for green determination of DPZ and CUR by the proposed HPLC-DAD method

Parameter	DPZ	CUR
Concentration (µg/ml)	0.1–100 µg/ml	0.1–100 µg/ml
LOD (µg/ml)	0.0044	0.026
LOQ (µg/ml)	0.0132	0.079
Correlation coefficient(r)	0.9999	0.9999
Slope	102,796	346,464
Intercept	170,303	160,970
Sy/x	294	562
Sa	136	2739
Sb	3.3	66.7
% Error	0.25	0.283
% RSD	0.614	0.695

S_y/x_ is the standard deviation of the residuals, S_a_ is the standard deviation of the intercept of regression line, S_b_ is the standard deviation of the slope of regression line, % Error =% RSD/√n.

### Accuracy

The degree to which the test findings closely resemble their true or theoretical value is known as accuracy [45]. In order to ensure the accuracy of the recommended method, six pure samples were investigated in triplicate using HPLC-DAD at concentrations of ( 0.1, 0.5, 1, 5, 10 and 100 µg/mL) for DPZ and CUR. The significant mean % recovery values attained using the concentrations estimated from the proper regression equations revealed the accuracy of the recommended approach as stated in Table 3.

### Precision

Precision of an analytical method is the degree of consistency between repeated measurements performed on the same sample under similar circumstances [45]. Three replicate determinations for each concentration within one day were used to assess the suggested method’s within-day (intra-day) precision at three concentration levels for each analyte. However, between-day (interday) precision was attained by repeatedly assessing three various concentrations for each of the cited drugs over three consecutive days. The established method’s enhanced precision has been verified by the low values of percentage relative standard deviation (%RSD) which were less than 2.0% indicating reasonable consistency, as illustrated in Table 4.

**Table 3:** Analysis of DPZ and CUR in their pure form by HPLC-DAD

Compound	Proposed Method				Comparison Method [46]
DPZ	Concentration taken (µg/ml)	Concentration found (µg/ml)	% found*		% found*
	0.1	0.0983	98.30		99.31
	0.5	0.4999	99.98		100.56
	1	1.0000	100.00		99.02
	5	5.0046	100.09		
	10	9.99	99.97		
	100	100.02	100.00		
Mean ± S. D				99.72 ± 0.69	99.63 ± 0.82
t-Test	0.219(2.365)**				
F-Test	1.130(5.786)**				
CUR	Concentration taken (µg/ml)	Concentration found (µg/ml)		Proposed Method %found*	Comparison Method % found* [47]
	0.1	0.0983		98.30	98.15
	0.5	0.4994		99.88	101.25
	1	1.0006		100.06	99.75
	10	4.9994		99.99	
	50	10.0025		100.03	
	100	99.9998		100.00	
Mean ± S. D				99.70 ± 0.54	99.38 ± 1.09
t-Test	0.014(4.303) **				
F-Test	4.159(5.786) **				

** The theoretical t and F values at (*P* = 0.05) are enclosed in parentheses [48].

**Table 4:** Accuracy and precision data for the simultaneous determination of DPZ and CUR by the proposed HPLC-DAD method

Drug	Nominal value	Within-day Precision			Between-day Precision		
	µg/mL	mean ± SD	RSD (%)	Er (%)	mean ± SD	RSD (%)	Er (%)
DPZ	1	99.54 ± 0.75	0.75	0.43	99.30 ± 0.95	0.95	0.55
	50	100.23 ± 1.82	1.81	1.05	99.83 ± 1.55	1.55	0.90
	100	99.73 ± 1.12	1.12	0.65	99.75 ± 1.54	1.54	0.89
CUR	1	99.68 ± 1.46	1.47	0.85	99.95 ± 1.26	1.26	0.73
	50	99.8 ± 1.66	1.66	0.96	99.87 ± 1.05	1.05	0.61
	100	100.75 ± 0.76	0.76	0.44	100.18 ± 1.29	1.30	0.75

### Selectivity and specificity

An analytical technique’s selectivity is its capability to generate response that is nearly only reliant on the intended analyte or analytes existing in the sample, neglecting the impact of other interferences [45]. Quantitative measurements of DPZ and CUR in a laboratory prepared nano liposomal formulation have been employed to assess the selectivity of the suggested methodology, the high percentage of recoveries and low SD values illustrate that the listed drugs can be analyzed without interference from additional excipients, as demonstrated in Table 5.

**Table 5:** Determination of DPZ and CUR in their laboratory prepared nanoliposome formulation using the suggested HPLC-DAD and refrence methods

Nanoliposome Formulation	Concentration Taken (µg/ml)	Concentration Found (µg/ml)	Suggested Method % Found*	Reference Method % Found*[46,47]
DPZ	1	0.998	99.8	99.30
	50	50.10	100.2	101.20
	100	98.80	98.8	98.30
Mean ± S. D			99.6 ± 0.72	99.60 ± 1.47
%RSD			0.72	1.48
t-test			2.353(3.182)** 3.804(19.00)**	
F-test				
CUR	1	1.0145	101.45	102.00
	50	49.8500	99.7	101.34
	100	100.4000	100.4	98.99
Mean ± S. D			100.52 ± 0.89	100.78 ± 1.58
%RSD			0.88	1.57
t-Test			0.249(2.776)** 3.226(19.00)**	
F-Test				

^**^ The theoretical t and F values at (*P* = 0.05) are enclosed in parentheses [48]

### Robustness

According to ICH Q2 R2 guidelines, Analytical procedures’ robustness indicates their ability to withstand slight but intentional alterations in methodology conditions while maintaining reliability throughout everyday use [45]. In the suggested method, constant response was achieved with minor variations performed in one parameter while maintaining others constant, such as pH of the buffer portion in mobile phase (3.2 ± 0.1), flow rate (1.5 ± 0.05 mL/min), temperature (38, 40, 42 °C) and detection wavelength (± 2 nm). The developed methodolgy demonstrated reliable and reproducible peak areas and retention time for both drugs. These adjustments failed to have meaningful impact on either drug’s observed responses (peak areas). Additionally, the retention times and resolution between the two drug peaks were not significantly impacted.

### System suitability parameters

A system suitability investigation was conducted to confirm the validity of the suggested approach. Adopting the USP Reference values [49], it was concluded that the obtained findings have been within the accepted standards for the system suitability parameters, as shown in Table 6.

**Table 6:** System suitability parameters for the separated compounds in this study

Drug	t_R_±SD (min)	Capacity factor (k′)	Theoretical plates (*N*)	Tailing factor	Selectivity (α)	Resolution (Rs)
DPZ	3.6 ± 0.1	1.77	5184	1.15		
CUR	6.5 ± 0.1	4.00	3969	0.94	2.26	6.3

### Method applications

#### Assay of DPZ/CUR in synthetic mixtures and lab-prepared nanoliposome dosage form

The approach provided was adopted to determine DPZ and CUR within their synthetic mixtures created in the lab with varying ratios. Table 7 illustrates how the suggested methodology has been accurate due to its superb % recovery and low SD values. Furthermore, The suggested chromatographic approach worked efficiently for assessing the cited drugs in their co-formulated nanoliposome laboratory made formulation, as presented in Table 5. The sample processing involved just one step of extraction using methanol, confirming the elimination of any interference from the dosage form additives which put attention on the feasibility of the recommended approach as an environmentally friendly one for analytes’ quality assessment.

### Statistical anaysis

The Student’s t-test and variance ratio F-test have been applied to statistically compare the recovery data from the developed HPLC-DAD methodology to the reported methods [46, 47]. There have been no statistically meaningful variations between the recoveries achieved using the reported approaches and the ones acquired by the proposed method, as shown in Table 5.

**Table 7:** Application of the suggested HPLC-DAD methodology to the analysis of DPZ-CUR synthetic mixture

	Concentration Taken (µg/ml)	Concentration Found (µg/ml)	Concentration Taken (µg/ml)	Concentration found (µg/ml)	%found^*^	%found^*^
Mix. No	DPZ	DPZ	CUR	CUR	DPZ	CUR
1	1	0.9980	1	1.015	99.8	101.45
2	50	50.1000	50	49.85	100.2	99.7
3	100	98.8000	100	100.40	98.8	100.4
Mean ± S.D					99.60 ± 0.72	100.52 ± 0.88
%RSD					0.72	0.88
%Error					0.42	0.51

### Greenness evaluation

As a subfield of green chemistry, GAC initially emerged in 2000, earning a lot of attention among analysts. It is concerned with investigating how analytical chemistry researchers could boost laboratory practices while mitigating their adverse effects on the environment. The goal is establish procedures that produce the least amount of pollution while maintaining maximum efficiency [50]. To assess how environmentally friendly these techniques are, researchers have employed a variety of tools and metrics, such as MOGAPI, AGREE, BAGI and the RGB model [51].

#### Analytical GREEnness metric (AGREE)

**Fig. 7:**
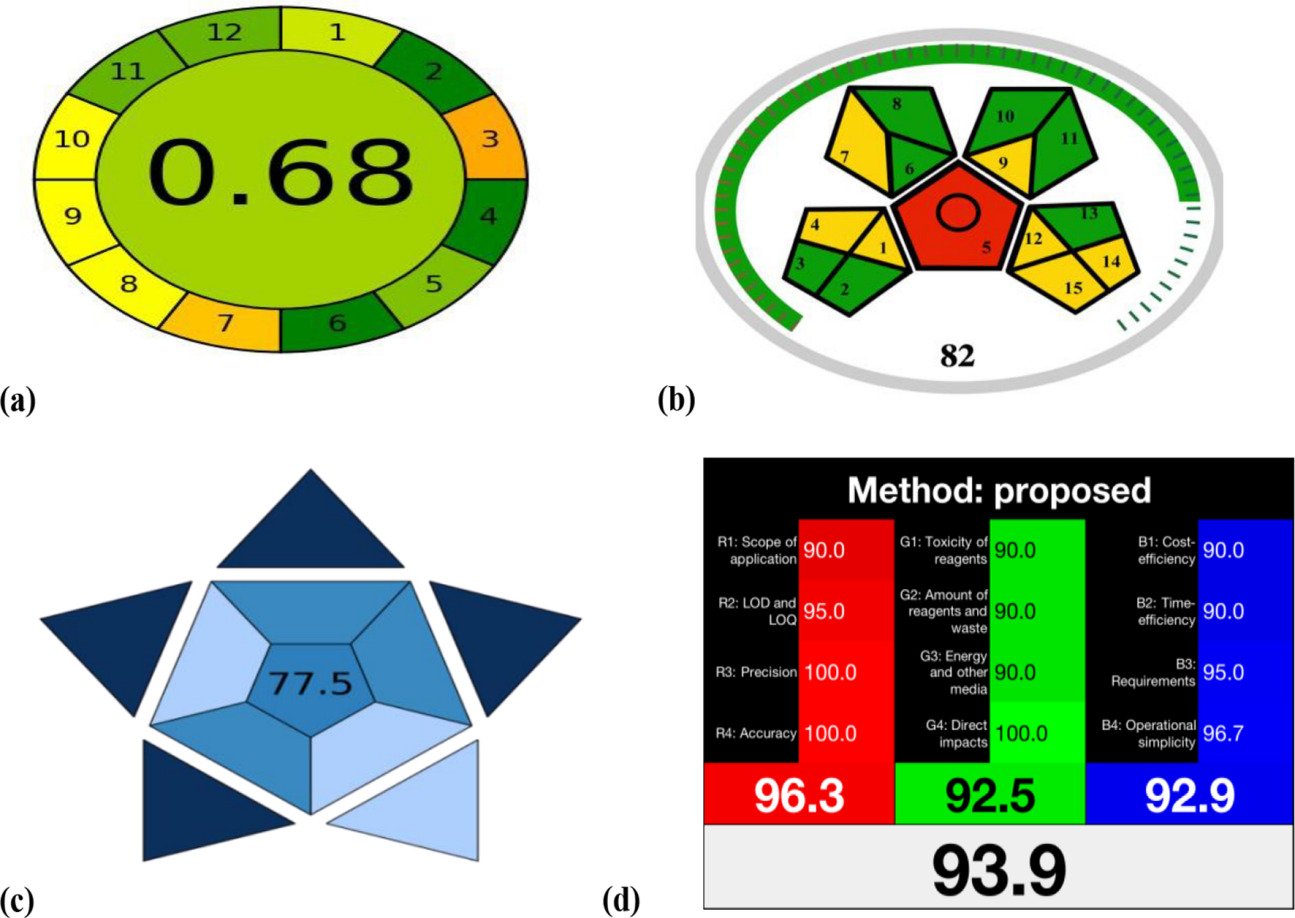
Assessment of the recommended HPLC method’s greenness via different metrics (**a**) MoGAPI Tool, (**b**) AGREE Tool, (**c**) BAGI Tool, (**d**) RGB Model

It is a freely downloadable greenness evaluation tool which was created by Pena-Pereira in June 2020 [37]. An easy-to-understand and informative result is produced by the AGREE calculator. The scoring parameters are derived from the 12 GAC principles (SIGNIFICANCE) and converted into a 0–1 scale. The resulting pictogram is divided into twelve sections, and each one’s width may be modified according to its prominence. The color of each section is different and varies from dark green (= 1) to dark red (= 0). The final score is shown in the centre of the spherical pictogram. AGREE calculated score of (0.68) illustrates that the developed HPLC-DAD method is environmentally begnin, as shown in Fig. 7(a).

#### Modified green analytical procedure index (MoGAPI)

For assessing the overall greenness of an analytical procedure, GAPI has shown to be a helpful semi-quantitative assessment tool in laboratory research which was created in 2018. It is composed of five pentagons involving 15 distinct elements starting from sample collection and preparation, waste handling, the effects that reagents and other solvents may have on analyst’s safety and instrumentation upto final analysis. Similar to traffic lights, GAPI uses a three-color scale for each step, with green designating a safe procedure, yellow for mild environmental or human health impact and red for those which are not ecologically friendly. Nevertheless, the GAPI measure does not provide a total score that would allow rapid comparison between various analytical procedures. Therefore, the MoGAPI tool was developed in 2024 that brings together the benefits of GAPI’s graphical design with an accurate overall score [38]. By applying MoGAPI to the developed method, it was observed that the chart’s overall score of 82 and the shades of the scale around the pentagrams reflects the method’s excellent greenness, as presented in Fig. 7(b).

#### Blue applicability grade index (BAGI)

An analytical method’s practicality is assessed using a novel assessment tool called BAGI. It is primarily concerned with the applications of WAC and may be regarded as a supplement to the widely recognised green metrics. An approach is deemed environmentally friendly and appropriate for everyday use if its BAGI index is at least 60 [33]. We further employed BAGI to guarantee a thorough evaluation and our approach received a score of 77.5 on the BAGI assessment. Figure 7(c). is demonstrating the proposed approach’s suitability and practicality in real-world applications.

#### Red–Green–Blue (RGB) model

White analytical chemistry is not only concerned with the method’s greenness but also with its usefulness relying on its analytical efficiency (red), which is measured by the validation criteria (accuracy, precision, and sensitivity), in addition to its practicality and financial factors (blue) like the method’s overall simplicity, speed, and cost [32]. The RGB 12 algorithm is used to assess the analytical approach’s whiteness. It is offered as an Excel sheet template that has to be filled up involving three tables: red, green, and blue. Our developped method has been proven to be white according to the concepts of WAC showing balance between sustainability, analytical performance and practicality, as shown in Fig. 7(d).

## Conclusion

In conclusion, the developed green HPLC-DAD method represents a significant advancement in environmentally conscious analytical techniques. By optimizing solvent selection, reducing hazardous waste, and improving overall energy efficiency, this method aligns with the principles of GAC and WAC while maintaining high sensitivity, accuracy, and reproducibility for the targeted analytes. The method’s applicability to analyse DPZ and CUR in the laboratory prepared nanoliposomal formulation and its capacity for rapid, reliable quantification, without interference from the excipients present, underscore its potential for widespread use in pharmaceuticals industry and quality control labs. Future work may focus on further minimizing solvent consumption and expanding the method’s utility across a broader range of complex samples, contributing to the ongoing efforts to make analytical chemistry more sustainable and eco-friendly.

## Electronic supplementary material

Below is the link to the electronic supplementary material.

Supplementary Material 1

## Data Availability

Data is provided within the manuscript or supplementary information files.

## Abbreviations

DPZ: Donepezil HCl
CUR: Curcumin
AD: Alzheimer’s disease
NFTs: Neurofibrillary tangles
BBB: Blood brain barrier
ICH: International Conference on Harmonization
HPLC: High performance liquid chromatography
HPTLC: High performance thin liquid chromatography
MoGAPI: Modified Green Analytical Procedure Index
AGREE: Analytical Greenness metric approach
GAC: Green Analytical Chemistry
WAC: White Analytical Chemistry
BAGI: Blue Applicability grade index
AchE: Acetylcholinesterase
BchE.: Butyrylcholinesterase
