# Supplementary material for: Assessment of greenness, blueness, and whiteness profiles of a validated HPLC-DAD method for quantitation of Donepezil HCl and Curcumin in their laboratory prepared co-formulated nanoliposomes
Source: BMC Chem. 2025 Jan 18;19(1):17. doi: 10.1186/s13065-024-01377-y (PMC11743032; doi:10.1186/s13065-024-01377-y)
Supplement: Supplementary file 1 — Supplementary Material 1 [file 13065_2024_1377_MOESM1_ESM.docx]

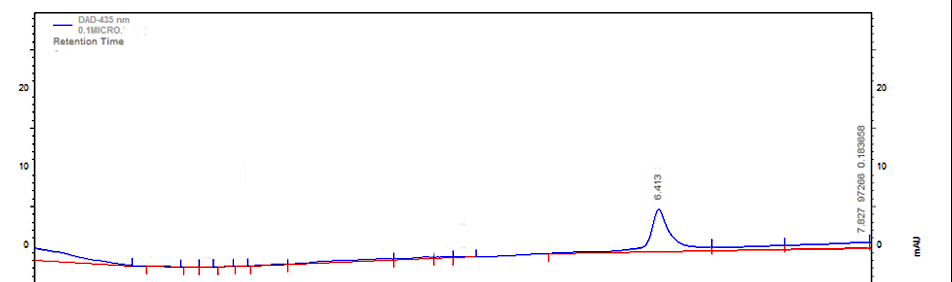


AUC


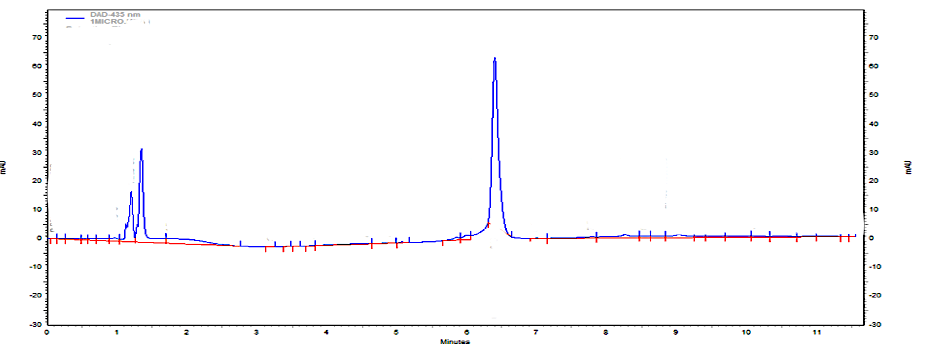


AUC


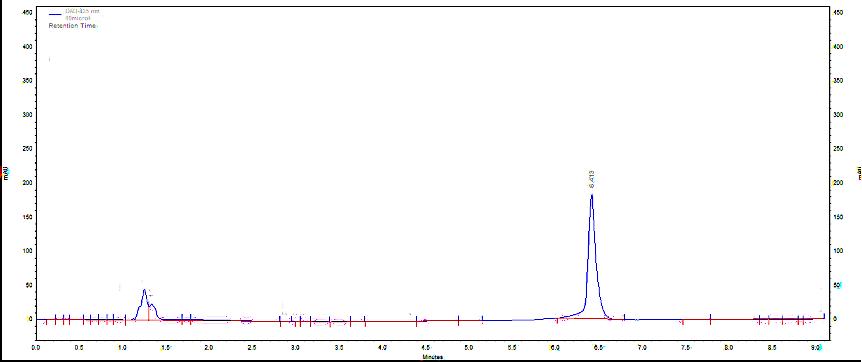

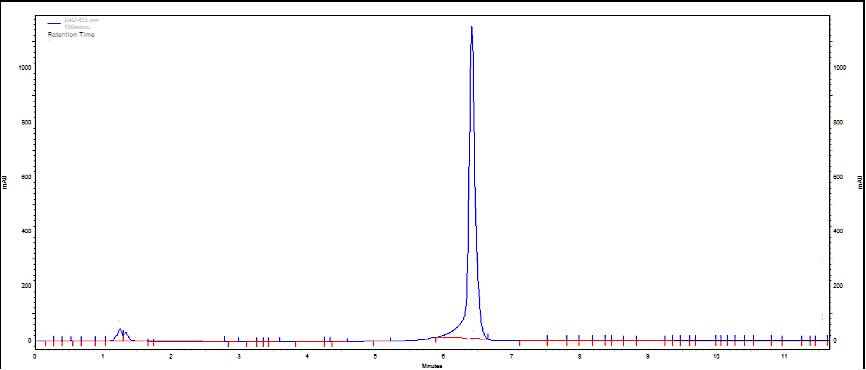


AUC

AUC

**FIGURE 5** Representative HPLC-DAD chromatogram for CUR (a) 0.1 µg/ml, (b) 1 µg/ml (c) 10 µg/ml and (d) 100 µg/ml (tr=6.6±0.2) at 415 nm.


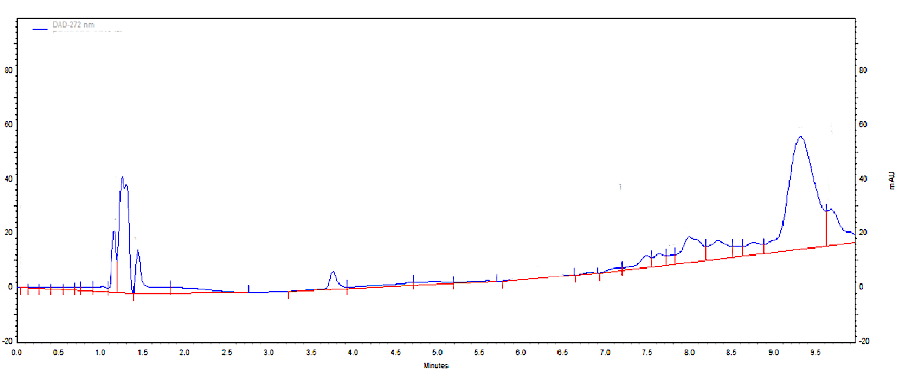

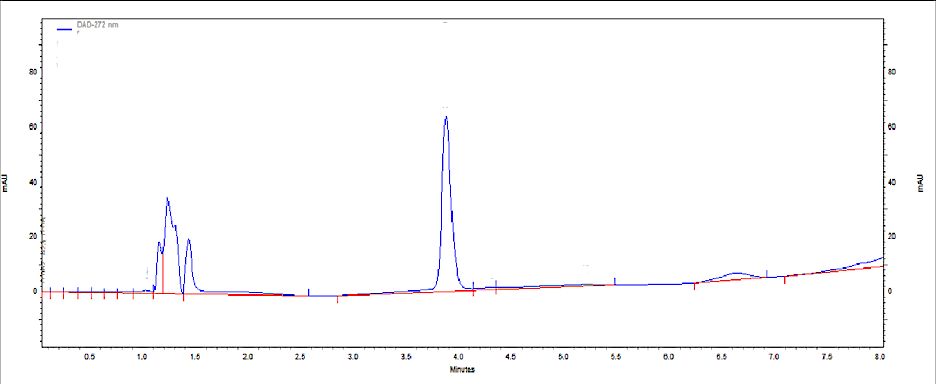


AUC

AUC

**
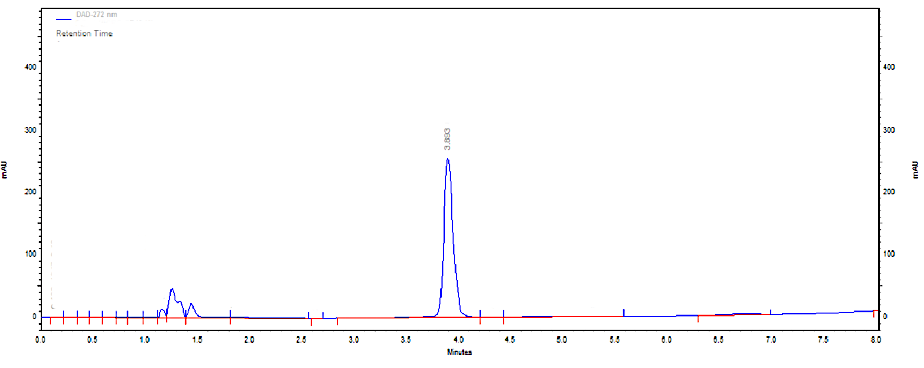
**

AUC

AUC

**
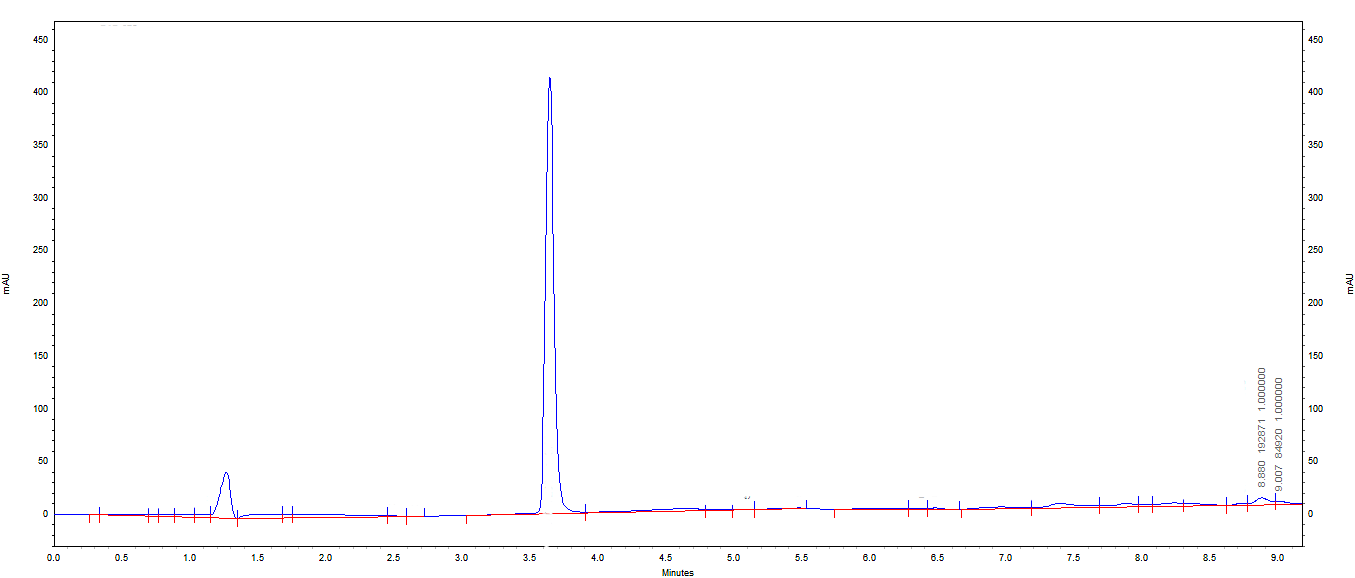
**

**FIGURE 6** Representative HPLC-DAD chromatogram for DPZ (a) 0.1 µg/ml, (b) 10 µg/ml (c) 50 µg/ml and (d) 100 µg/ml (tr=3.6±0.2) at 273nm
